# Roux-en-Y Gastric Bypass and Sleeve Gastrectomy Risk-Adjusted Safety Profiles after Previous Fundoplication: A 499-Patient MBSAQIP Analysis

**DOI:** 10.1007/s11695-025-08404-4

**Published:** 2025-11-22

**Authors:** Pattharasai Kachornvitaya, Valentin Mocanu, Mélissa V. Wills, Juan S. Barajas-Gamboa, Salvador Navarrete, Ricard Corcelles, Andrew Strong, Suthep Udomsawaengsup, Matthew Kroh, Jerry Dang

**Affiliations:** 1 Digestive Disease Institute, Cleveland Clinic, Ohio, Cleveland, USA; 2 Cleveland Clinic Lerner College of Medicine of Case Western Reserve University, Cleveland, USA; 3 Digestive Diseases Institute, Cleveland Clinic Abu Dhabi, Abu Dhabi, United Arab Emirates; 4 Department of Surgery, Faculty of Medicine, Chulalongkorn University, Bangkok, Thailand; 5 Treatment of Obesity and Metabolic Disease Research Unit, Faculty of Medicine, Chulalongkorn University, Bangkok, Thailand

**Keywords:** Revisional bariatric surgery, Fundoplication, Sleeve gastrectomy, Roux-en-Y gastric bypass, Gastroesophageal reflux disease

## Abstract

**Background:**

Obesity and gastroesophageal reflux disease (GERD) frequently coexist and may necessitate surgical intervention when conservative management fails. While fundoplication is effective for GERD, many patients ultimately require metabolic and bariatric surgery (MBS) for obesity. The safety and prevalence of sleeve gastrectomy (SG) or Roux-en-Y gastric bypass (RYGB) after prior fundoplication remains poorly characterized.

**Objectives:**

To assess the prevalence and 30-day serious complication rates of SG and RYGB following fundoplication, and to identify independent predictors of adverse outcomes.

**Methods:**

A retrospective analysis of the 2022–2023 MBSAQIP database identified 499 patients undergoing primary SG or RYGB after fundoplication. Outcomes assessed included leak, bleeding, reoperation, reintervention, cardiac events, pneumonia, kidney injury, thromboembolism, infection, or sepsis. Multivariable logistic regression was used to adjust for baseline differences.

**Results:**

Of 499 patients, 126 (25.3%) underwent SG (F-SG) and 373 (74.7%) underwent RYGB (F-RYGB). Concomitant paraesophageal hernia repair was more common in the F-RYGB group (54.2% vs. 26.2%, *p* < 0.001). Mean BMI was higher in F-SG (42.4 vs. 40.1 kg/m², *p* < 0.001). Weight gain and GERD were the leading indications for revisional bariatric surgery. F-RYGB was associated with longer operative times, hospital stay, and higher rates of reoperation, readmission, bowel obstruction, and serious complications (9.6% vs. 4.0%, *p* = 0.045). Rates of anastomotic leak, postoperative bleeding, and composite wound complications were low but numerically higher in F-RYGB, without statistical significance. In multivariate analysis, procedure type was not significantly associated with serious complications (OR 2.03, 95% CI 0.68–6.09, *p* = 0.207).

**Conclusions:**

Both SG and RYGB appear to be safe and effective revisional options, and a history of fundoplication should not be considered a contraindication. Although RYGB was associated with longer operative times and higher early complication rates, multivariable analysis showed no significant difference in serious complications between the two procedures.

## Highlights

- 74.7% had RYGB; 25.3% underwent SG after failed fundoplication.
- Main indications were weight gain (43.1%) and GERD (32.1%).
- Unadjusted complications higher in RYGB vs. SG (9.6% vs. 4.0%, *p* = 0.045).
- After adjustment, RYGB was not a significant predictor of complications.

## Introduction

 Gastroesophageal reflux disease (GERD) and obesity frequently coexist, each contributing to the severity and progression of the other. Obesity increases intra-abdominal pressure and promotes reflux, while GERD may be worsened by anatomical and physiological changes associated with excess weight. Fundoplication remains the most common surgical treatment for medically refractory GERD, with long-term success rates up to 90% reported in non-obese populations [1]. However, failure rates can approach 30%, particularly in patients with obesity due to factors such as wrap disruption and migration, recurrent hiatal hernia, or persistent dysphagia [2, 3]. These high failure rates present a significant clinical challenge, necessitating clear guidance on optimal revisional approaches.

When fundoplication fails, revisional surgery may be required. Often, redo fundoplication is the most common approach; however, Roux-en-Y gastric bypass (RYGB) has gained traction as a viable alternative. RYGB addresses both reflux and obesity through anatomical diversion and reduction of gastric acid exposure and may help address gastric motility disorders. Recent systematic reviews have demonstrated high symptom resolution rates (70–93%) and substantial weight loss after RYGB in this setting, with acceptable perioperative morbidity and low mortality [4–6]. Moreover, RYGB has been associated with a lower recurrence of reflux symptoms compared to redo fundoplication in patients with obesity as a low pressure, parietal separating procedure [5, 6]. This dual benefit of addressing both weight and reflux makes RYGB particularly appealing for this patient population.

Sleeve gastrectomy (SG), while increasingly popular as a primary bariatric procedure, has historically been considered less favorable following antireflux surgery due to its potential to exacerbate GERD. Some evidence suggests that SG can be safely performed after prior fundoplication in selected patients, achieving reasonable outcomes [7, 8]. However, its long-term efficacy in managing GERD, addressing complications, and treating obesity remains a major concern, including increased rate of GERD from a relatively high pressure gastric tube, for which RYGB is most recommended. Despite these findings, comparative safety and outcomes data on SG and RYGB in the revisional context remain limited, especially in large, multicenter cohorts.

To address this gap, we conducted a retrospective analysis using the Metabolic and Bariatric Surgery Accreditation and Quality Improvement Program (MBSAQIP) database. This study aimed to evaluate the prevalence, perioperative safety, and predictors of serious complications in patients undergoing SG or RYGB after previous fundoplication, to better inform surgical decision-making and patient counseling in this complex and growing patient population. We compare outcomes between these procedures to provide evidence-based guidance for selecting the optimal surgical approach that balances safety with effectiveness.

## Methods

### Data Source and Ethical Approvals

A retrospective analysis of the MBSAQIP data registry was performed between the year 2022 and 2023. The MBSAQIP currently captures clinical data from nearly 1,000 accredited American and Canadian centers. The data registry prospectively collects data and contains standardized pre-, intra-, and post-operative variables specific to bariatric surgery patients. We restricted our analysis to these years because the MBSAQIP database began recording fundoplication as a prior procedure only after 2022. This study used de-identified data and was exempt from Institutional Review Board approval according to institutional policies.

## Patient Variables and Population

All patients who underwent SG or RYGB with previous history of fundoplication between 2022 and 2023 were included in this retrospective cohort study. We limited our analysis to these years because fundoplication was only captured as a previous procedure in the MBSAQIP database after 2022.

The initial cohort was identified using the *conv_prev_proc* variable if cases were coded as receiving prior fundoplication. However, the dataset lacked details regarding the specific types of fundoplication performed and how the procedure was managed intraoperatively during bariatric surgery. After identifying this cohort, only cases coded as SG or RYGB were included using the *conv_curr_proc* variable. Indications for either revision or conversion were categorized using the *rev_conv_finindic* variable.

Basic demographic data including age, sex, race, body mass index (BMI), functional status, American Society of Anesthesiologists (ASA) Physical Status classification and smoking status within the past year were collected. Patient-associated medical problems included the following: type 2 diabetes, hypertension, GERD, chronic obstructive pulmonary disease (COPD), hyperlipidemia, obstructive sleep apnea, renal insufficiency, venous stasis, and preoperative therapeutic anticoagulant use. Patient history included previous venous thromboembolism (VTE), myocardial infarction (MI), percutaneous coronary intervention, and major cardiac surgery. All variables were defined according to the standardized MBSAQIP data dictionary definitions.

## Objectives

The primary objective of this study was to determine the rate of serious complications and mortality of SG and RYGB after fundoplication. Serious complications were defined as the occurrence of at least one of the following events within 30 days of surgery: anastomotic leak, postoperative bleeding, reoperation, non-operative intervention, cardiac event (cardiac arrest, myocardial infarction, or cardiopulmonary resuscitation), pneumonia, unplanned intubation, acute kidney injury, venous thromboembolism, composite wound complications (superficial and deep surgical site infection, and wound disruption), sepsis, or cerebrovascular accident.

We aimed to develop a multivariable logistic regression model to determine risk factors associated with serious complications and mortality for patients undergoing SG and RYGB after fundoplication. Variables were selected for inclusion in the multivariable model based on clinical relevance and statistical significance in univariate analysis (*p* < 0.05). Secondary objectives included identifying and characterizing indications for SG and RYGB after fundoplication.

### Statistical Analysis

Categorical variables were summarized as percentages, while continuous variables were reported as means ± standard deviations (SD). Baseline differences between groups were assessed using chi-squared tests for categorical variables and independent sample t-tests for continuous variables. To compare outcomes between the SG and RYGB cohorts, univariate logistic regression analyses were conducted. Multivariable logistic regression was then used to identify independent predictors of serious complications and 30-day mortality. Variables with a p-value < 0.05 in univariate analysis were included in the multivariable model. Patient characteristics and operative time were also incorporated. Collinearity among variables was assessed using variance inflation factors (VIFs), with values > 5 considered indicative of significant collinearity. Model performance was evaluated using the area under the receiver operating characteristic (AUROC) curve for discrimination, and the Brier score for calibration. An AUROC of > 0.7 was considered acceptable discrimination, while a Brier score < 0.1 indicated good calibration. Missing data were handled using the available case method, as all variables had less than 5% missingness. Sensitivity analyses were performed to assess the robustness of findings to alternative modeling approaches. All statistical analyses were completed using STATA 17 statistical software (StataCorp, College Station, TX, USA).

## Results

### Patient Baseline Characteristics

A total of 499 patients, 126 (25.3%) patients underwent SG after fundoplication (F-SG) and 373 (74.7%) patients underwent RYGB after fundoplication (F-RYGB). The baseline characteristics are summarized in Table 1. The mean age was similar between the two groups (F-SG: 52.3 ± 10.6 years vs. F-RYGB: 53.6 ± 9.9 years, *p* = 0.2). A higher proportion of females underwent F-RYGB compared to F-SG, although this was not statistically significant (*p* = 0.051). Patients in the F-SG group had a significantly higher body mass index (BMI) (42.4 ± 5.9 kg/m² vs. 40.1 ± 6.5 kg/m², *p* < 0.001). GERD was significantly more common in the F-RYGB group (73.7% vs. 47.6%, *p* < 0.001). This substantial difference in GERD prevalence likely reflects surgical decision-making, with RYGB preferentially selected for patients with more severe reflux symptoms. There were no significant differences in other associated medical problems, including diabetes, hypertension, dyslipidemia, obstructive sleep apnea, chronic obstructive pulmonary disease, or renal insufficiency, between the groups.

**Table 1:** Baseline characteristics of patients undergoing bariatric surgery after fundoplication

Characteristic	F-SG (*n* = 126)	F-RYGB (*n* = 373)	*p*-value
Age (years, mean ± SD)	52.3 ± 10.6	53.6 ± 9.9	0.194
Female, n (%)	98 (77.8)	318 (85.3)	0.051
Race, n (%)			0.012
White	89 (70.6)	307 (82.3)	
Black or African American	23 (18.3)	35 (9.4)	
Other	14 (11.1)	31 (8.3)	
Body mass index (kg/m2, mean ± SD)	42.4 ± 5.9	40.1 ± 6.5	< 0.001
Functional Status, n (%)			0.445
Independent	124 (98.4)	370 (99.2)	
Partially Dependent	2 (1.6)	3 (0.8)	
ASA Category, n (%)			0.500
ASA 1–2	15 (11.9)	60 (16.1)	
ASA 3	106 (84.1)	301 (80.7)	
ASA 4–5	5 (4)	12 (3.2)	
Smoker in the past year, n (%)	7 (5.6)	18 (4.8)	0.745
Diabetes, n (%)			0.781
None or Diet Controlled	92 (73)	264 (70.8)	
Non-insulin Dependent	25 (19.8)	75 (20.1)	
Insulin Dependent	9 (7.1)	34 (9.1)	
Hypertension, n (%)	81 (64.3)	206 (55.2)	0.075
Gastroesophageal reflux disease, n (%)	60 (47.6)	275 (73.7)	< 0.001
Dyslipidemia, n (%)	42 (33.3)	155 (41.6)	0.103
Obstructive sleep apnea, n (%)	66 (52.4)	178 (47.7)	0.366
Chronic obstructive pulmonary disease, n (%)	5 (4)	14 (3.8)	0.913
Immunosuppressive therapy, n (%)	7 (5.6)	19 (5.1)	0.840
Renal Insufficiency, n (%)	1 (0.8)	3 (0.8)	0.991
History of venous thromboembolism	8 (6.4)	24 (6.4)	0.973
Venous stasis, n (%)	1 (0.8)	4 (1.1)	0.786
Therapeutic anticoagulation use, n (%)	9 (7.1)	28 (7.5)	0.893
History of myocardial infarction, n (%)	5 (4)	10 (2.7)	0.464
Previous percutaneous coronary intervention, n (%)	2 (1.6)	2 (0.5)	0.253
Previous major cardiac surgery, n (%)	6 (4.8)	7 (1.9)	0.079

Abbreviations: *F-SG,* Fundoplication to sleeve gastrectomy; *F-RYGB,* fundoplication to Roux-en-Y gastric bypass; *RYGB,* Roux-en-Y gastric bypass; *SG,* Sleeve gastrectomy; *ASA,* American Society of Anesthesiologists

## Indications for Bariatric Surgery after Fundoplication

Table 2 outlines the clinical indications prompting conversion to bariatric surgery. The most common indication was weight gain, accounting for 43.1% of cases. This may represent patients seeking bariatric surgery primarily for weight management rather than failed reflux control from their original fundoplication. The second most common indication was GERD (32.1%), suggesting persistent or recurrent reflux after fundoplication. Other less frequent indications included persistent associated medical problems (4.81%), dysphagia (1.4%), adhesions (0.8%), nausea and/or vomiting (0.6%), abdominal pain, gastrointestinal tract ulcer, and patient intolerance, each contributing to 0.2% of cases. An additional 16.6% of cases were categorized under other causes not specifically defined.

**Table 2:** Indications for bariatric surgery after fundoplication

Indications *n* (%)	
Weight gain	215 (43.1)
Gastroesophageal reflux disease	160 (32.1)
Persistent associated medical problems	24 (4.81)
Dysphagia	7 (1.4)
Adhesions	4 (0.8)
Nausea and/or vomiting	3 (0.6)
Abdominal pain	1 (0.2)
Patient intolerance	1 (0.2)
Gastrointestinal tract ulcer	1 (0.2)
Others	83 (16.6)

## Procedural Details and Postoperative Complications

Concomitant paraesophageal hernia repair was significantly more common in the F-RYGB group compared to the F-SG group (54.2% vs. 26.2%, *p* < 0.001). The operative length was significantly shorter in the F-SG group (90.6 ± 67.1 min) compared to the F-RYGB group (200 ± 89.1 min, *p* < 0.001). Additionally, the length of hospital stay was significantly shorter in the F-SG group (1.3 ± 0.8 days vs. 2 ± 1.9 days, *p* = 0.001).

Regarding postoperative complications, the F-RYGB group experienced a significantly higher rate of serious complications compared to the F-SG group (9.6% vs. 4.0%, *p* = 0.045). Specific complications that were significantly more common in the F-RYGB group included reoperation (4.0% vs. 0%, *p* = 0.022), readmission (11.3% vs. 3.2%, *p* = 0.007), and bowel obstruction (3.2% vs. 0%, *p* = 0.042). There were no statistically significant differences in rates of anastomotic leak (1.1% vs. 0.8%, *p* = 0.786), postoperative bleeding (2.4% vs. 0%, *p* = 0.078), composite wound complications (4.3% vs. 1.6%, *p* = 0.160), sepsis, unplanned intubation, non-operative intervention, cardiac events, pneumonia, acute kidney injury, venous thromboembolism, or mortality between the two groups (Table 3).

**Table 3:** Perioperative details and 30-day postoperative complications

	F-SG (*n* = 126)	F-RYGB (*n* = 373)	*p*-value
Operative details			
Concomitant paraesophageal hernia repair, n (%)	33 (26.2)	202 (54.2)	< 0.001
Operative time (mins), mean ± SD	90.6 ± 67.1	200 ± 89.1	< 0.001
Laparoscopic approach, n (%)	125 (99.2)	372 (99.7)	0.192
Length of stay (days), mean ± SD	1.3 ± 0.8	2 ± 1.9	0.001
Surgical complications, n (%)			
Leaks	1 (0.8)	4 (1.1)	0.786
Postoperative bleeding	0 (0)	9 (2.4)	0.078
Reoperation	0 (0)	15 (4)	0.022
Non-operative intervention	2 (1.6)	15 (4)	0.193
Readmission	4 (3.2)	42 (11.3)	0.007
Cardiac events	0 (0)	4 (1.1)	0.243
Pneumonia	1 (0.8)	4 (1.1)	0.786
Bowel obstruction	0 (0)	12 (3.2)	0.042
Infectious complications, n (%)			
Composite wound complications	2 (1.6)	16 (4.3)	0.160
Superficial SSI	0 (0)	5 (1.3)	0.191
Deep SSI	2 (1.6)	10 (2.7)	0.488
Wound Disruption	0 (0)	1 (0.3)	0.561
Sepsis	0 (0)	2 (0.5)	0.410
Medical complications, n (%)			
Unplanned intubation	0 (0)	3 (0.8)	0.313
Venous thromboembolism	0 (0)	6 (1.6)	0.152
Acute kidney injury	1 (0.8)	0 (0)	0.085
Composite outcomes, n (%)			
Serious complications	5 (4)	36 (9.6)	0.045
Mortality	0 (0)	2 (0.5)	0.410

Abbreviations: *F-SG*, Fundoplication to sleeve gastrectomy; *F-RYGB*, Fundoplication to Roux-en-Y gastric bypass;* SSI*, Surgical site infection

###  Multivariable Logistic Regression for Predictors of Serious Complications 

After adjustment using multivariable logistic regression, the comparison between surgical procedures showed no statistically significant difference between F-RYGB and F-SG. (OR 2.03, 95% CI: 0.68–6.09, *p* = 0.207). Other factors evaluated, including concomitant paraesophageal hernia repair, higher BMI, older age, female sex, race, diabetes status, hypertension, GERD, history of VTE, therapeutic anticoagulant use, and longer operative times, were not significantly associated with serious complications. This serious complication model had an AUROC of 0.69 and Brier score of 0.07, indicating acceptable discrimination and good calibration (Table 4).

**Table 4:** Predictors of serious complications after bariatric surgery following fundoplication

	Adjusted odds ratio^*^	95% Confidence Interval	*p*-value
Concomitant paraesophageal hernia repair	0.86	0.43–1.73	0.679
Higher BMI (per 5 kg/m^2^)	1.18	0.92–1.52	0.188
Older age (per 10 years)	0.84	0.57–1.21	0.342
Female	0.67	0.28–1.63	0.376
Race			
Black	1.14	0.41–3.23	0.799
Other	0.85	0.24–3.01	0.806
Functional status	6.76	0.84–54.38	0.072
Diabetes			
Non-insulin dependent	0.84	0.34–2.06	0.701
Insulin dependent	1.23	0.38–4.02	0.728
Hypertension	1.07	0.52–2.24	0.847
GERD	2.06	0.87–4.86	0.098
History of VTE	1.85	0.56–6.17	0.315
Therapeutic anticoagulant	1.66	0.51–5.43	0.399
F-RYGB vs. F-SG	2.03	0.68–6.09	0.207
Longer operative times	1.00	0.99–1.00	0.359

Abbreviations: *F-SG,* Fundoplication to sleeve gastrectomy; *F-RYGB*, fundoplication to Roux-en-Y gastric bypass; *BMI*, body mass index; *GERD*, Gastroesophageal reflux disease; *VTE*, Venous thromboembolism

## Discussion

This study represents one of the largest analyses using the MBSAQIP database to evaluate perioperative outcomes of revisional bariatric surgery following prior fundoplication, directly comparing SG and RYGB. Although unadjusted analyses demonstrated a higher rate of serious complications among patients undergoing RYGB, this association was not statistically significant after adjustment for baseline comorbidities and operative factors. The overall 30-day serious complication rate in this cohort was 8.2%, aligning with prior studies reporting increased morbidity in revisional procedures compared to primary bariatric operations.

Our findings are consistent with prior literature, which demonstrates that revisional surgery after failed fundoplication is associated with increased morbidity compared to primary procedures. While SG has historically been considered relatively contraindicated in patients with GERD due to concerns about worsening reflux, Osan et al. reported that SG is feasible in patients with prior antireflux repair, demonstrating low morbidity and comparable excess weight loss to standard SG procedures [6]. Recent evidence suggests it can be safely performed and possibly without takedown of the previous fundoplication. Kassir et al. conducted a prospective study indicating that SG can be successfully performed without dismantling the fundoplication, offering a bariatric option for patients with prior antireflux surgery [7]. SG provides a technically less complex alternative to RYGB, making it suitable for high-risk or anatomically challenging cases, particularly in patients without significant preoperative reflux or when RYGB is contraindicated, but need carefully selected patients.

Coakley et al. reported a 12.6% recurrence rate of reflux symptoms and an 80.4% excess weight loss following RYGB after failed fundoplication, with 9.2% requiring reoperation during follow-up, indicating acceptable long-term outcomes despite procedural complexity [8]. Similarly, Ibele et al. found that while complication rates were higher in patients undergoing RYGB after fundoplication (36% vs. 7%), weight loss outcomes were comparable to primary RYGB [9]. A meta-analysis by Bhat et al. also supports our results, showing a pooled morbidity of 39.5% for RYGB in patients post-fundoplication, though our observed complication rates were considerably lower. Bhat et al. also reported significantly better weight loss and found to have even greater levels of satisfaction compared to redo fundoplication [4]. Although not statistically significant, our cohort demonstrated that RYGB group exhibited higher rates of serious complications including leak, bleeding, and wound complications. These represent clinically important major events that may be attributed to the greater technical complexity of the procedure. However, these data suggest that while RYGB carries higher perioperative risk, longer operative times, and greater hospitalization requirements, it provides sustained benefits in weight reduction and reflux control. In appropriately selected patients, particularly those with obesity or recurrent GERD, these advantages may outweigh the short-term risks.

When comparing revisional techniques, there is no strong evidence directly comparing SG and RYGB; however, several studies have highlighted the potential benefits of RYGB over redo fundoplication, particularly in patients with obesity. Giulini et al. noted that RYGB, when performed early in the disease course, can achieve outcomes comparable to redo fundoplication with similar perioperative morbidity [10]. Likewise, Castillo-Larios et al. found RYGB to offer superior reflux symptom resolution compared to redo fundoplication (89.5% vs. 64.6%) and identified RYGB as a protective factor against persistent reflux in multivariable analysis [11]. The reoperation rates for RYGB after failed fundoplication are typically around 10–11% in most studies, including our cohort. This is attributed to procedure-specific complications, which occur more frequently following RYGB [12, 13]. Recent evidence from a national cohort study in France demonstrated that patients with a history of prior fundoplication had a higher rate of postoperative complications compared to those without fundoplication. Abboretti et al. showed that prior fundoplication was significantly associated with an increased risk of complications following SG (OR 2.54, 95% CI 1.37–4.32, *p* < 0.001), whereas the association with RYGB showed similar trend but did not reach statistical significance (OR 1.71, 95% CI 1.00–2.74, *p* = 0.051) [14].

Concurrent paraesophageal hernia (PEH) are frequently identified during revisional surgery for failed fundoplication, particularly in patients who underwent RYGB. A concurrent PEH was present in 85.1% of patients undergoing RYGB following failed fundoplication, which is almost 40% higher than in our cohort and is a common cause of fundoplication failure, often requiring revisional surgery [8]. PEH repair adds operative complexity due to scar tissue, altered anatomy, wrap migration, and entails greater technical demands and higher short-term morbidity (reported rates 18–39%) than primary bariatric or anti-reflux procedures [4, 15]. Some studies have evaluated RYGB without takedown of an intact wrap, which may reduce operative time and risk but potentially compromise weight loss and symptom relief, including longer term GERD symptoms [7, 16]. When combined with PEH repair, RYGB offers superior reflux control and reduced hernia recurrence, particularly in patients with obesity or complex cases. Early conversion to RYGB may improve outcomes compared to repeat fundoplication. However, concomitant PEH repair during bariatric surgery in patients with previous fundoplication has not been identified as an independent risk factor for serious complications.

Current guidelines remain inconclusive regarding the preferred revisional strategy after failed fundoplication. The findings of this study support the overall safety of both SG and RYGB in patients with a prior history of fundoplication undergoing bariatric surgery. While the RYGB group exhibited a higher incidence of complications compared with the SG group, this difference did not reach statistical significance. A prior fundoplication should not be considered a contraindication. RYGB may entail increased intraoperative difficulty and early postoperative complications, these risks can be mitigated through proper patient selection and execution in high-volume centers with multidisciplinary expertise. In our cohort, RYGB was the preferred surgical approach in patient with prior fundoplication, utilized in 74.7% of cases, particularly among patients with persistent obesity and GERD.

In fact, recent studies suggest that RYGB should be considered earlier in the treatment course for patients with obesity and severe reflux disease, rather than being reserved solely as a rescue option [5, 10]. RYGB addresses both the underlying GERD and obesity, with diversion of acid through separation of parietal cells. Additionally, patients with vagus nerve injury or gastric dysmotility may benefit from Roux reconstruction [17]. Thus RYGB may offer more durable symptom control and greater metabolic benefits [18].

The choice between SG and RYGB in this setting should be tailored to the individual patient rather than safety profiles alone. Given the distinct risk-benefit profiles of each procedure, careful preoperative evaluation and counseling are critical. A multidisciplinary approach that considers the indication for revision, patient anatomy, symptomatology, associated medical conditions, and surgeon expertise helps optimize outcomes and ensure appropriate procedure selection in revisional bariatric surgery following prior fundoplication.

The strength of this study lies in its use of a large, prospectively maintained national database (MBSAQIP), which allows for a broad assessment of perioperative outcomes in a real-world setting. Previous studies are limited to single and multi-center retrospective databases and may not reflect the broader surgical practice in North America. Nevertheless, the study is limited by its retrospective design and lack of long-term follow-up beyond 30 days, which restricts conclusions about sustained symptom resolution and weight loss durability. Additionally, we were unable to assess the severity of GERD symptoms before and after surgery, which would provide valuable information about the effectiveness of each procedure for symptom control, including objective studies including esophagitis, pH and motility studies. Furthermore, specific details, such as unknown indications for surgery, the type of prior fundoplication, whether it was taken down (particularly in SG patients), the technique used in paraesophageal hernia repair, the interval between fundoplication and bariatric surgery, anatomical findings at reoperation, and surgeon experience, were not available in the dataset. We also lacked information on specific institutional protocols for patient selection, which could introduce selection bias. Despite these limitations, the study provides robust evidence supporting the safety of both procedures as a revisional approach after fundoplication in the largest dataset available currently. Future prospective studies comparing both procedures with long-term follow-up and objective outcomes are needed to assess the durability of symptom relief and weight loss outcomes.

## Conclusion

Weight gain and GERD were the most common indications for revisional bariatric surgery after prior fundoplication. While RYGB was associated with longer operative times and higher rates of early complications, multivariable analysis demonstrated that procedure type was not significantly associated with serious complications. Both SG and RYGB are viable revisional options and prior fundoplication should not be considered a contraindication. Further prospective studies are warranted to assess long-term outcomes, particularly regarding durability of weight loss and reflux symptom control.

## Data Availability

No datasets were generated or analysed during the current study.
